# A Review of Childhood Developmental Changes in Attention as Indexed in the Electrical Activity of the Brain

**DOI:** 10.3390/brainsci14050458

**Published:** 2024-05-01

**Authors:** Sirel Karakaş

**Affiliations:** Psychology Department, Doğuş University, İstanbul 34775, Turkey; sirel@neurometrika-tech.com

**Keywords:** typically developing children, development of attention, clinical model of attention, connectivity, oscillatory dynamics, event-related brain activity, resting electroencephalogram

## Abstract

This review aims to present age-related changes in the neuroelectric responses of typically developing children (TDC) who are presumed to meet developmental stages appropriately. The review is based on findings from the frequently used neuropsychological tasks of active attention, where attention is deliberately focused versus passive attention where attention is drawn to a stimulus, facilitatory attention, which enhances the processing of a stimulus versus inhibitory attention, which suppresses the processing of a stimulus. The review discusses the early and late stages of attentional selectivity that correspond to early and late information processing. Age-related changes in early attentional selectivity were quantitatively represented in latencies of the event-related potential (ERP) components. Age-related changes in late attentional selectivity are also qualitatively represented by structural and functional reorganization of attentional processing and the brain areas involved. The purely bottom-up or top-down processing is challenged with age-related findings on difficult tasks that ensure a high cognitive load. TDC findings on brain oscillatory activity are enriched by findings from attention deficit hyperactivity disorder (ADHD). The transition from the low to fast oscillations in TDC and ADHD confirmed the maturational lag hypothesis. The deviant topographical localization of the oscillations confirmed the maturational deviance model. The gamma-based match and utilization model integrates all levels of attentional processing. According to these findings and theoretical formulations, brain oscillations can potentially display the human brain’s wholistic–integrative functions.

## 1. Introduction

This review focuses on the typical development of attention due to its impact on almost all other cognitive functions within the human information processing system. Among numerous external or internal sources, selecting those the information processing system will treat is a function of attention. Complex modulation of the selectivity and/or the intensity of cognitive functions (for example, the strength of learning and the amount of information retained) at different stages of the system is another function of attention [1,2,3]. However, this extensive impact has also made attention an influential confounding variable for studies on other cognitive functions. Attention has a deterministic (as an independent variable) or, if not duly controlled, a confounding effect, irrespective of whether a behavioral approach or a cognitive neuroscience approach is employed. Consequently, any discourse on life span or age-related changes in brain activity should consider attention, even when the study is on other cognitive functions [4,5].

The way age influences attention is a crucial point that needs to be addressed before treating the issue of age-related changes in attentional processing. Ridderinkhof and van der Stelt [5] used between-channel selection and channel interference tasks to study the effect of age on event-related potentials (ERPs), which are associated with attention (N2 and P300). Two of the research hypotheses were overruled. One of the hypotheses assumed a global and undifferentiated age effect on all attention-related components, and the other assumed an age effect on the earliest component, which then propagated to the later one. The third hypothesis, which assumed an independent and differential effect of age on parameters of attention-related components, was confirmed. This finding justifies the present review on age-related changes in attentional processing.

## 2. Stages of Attentional Processing

The primary function of attention is selection. Attention helps to select environmental (external or internal) stimuli with specific properties, focus on the relevant stimulus, and sustain this focus. Attentional selectivity occurs at an early and late stage of information processing. Table 1 provides a summary, and Section 2.1 and Section 2.2 provide information on stages of selectivity, the tasks by which these stages are experimentally produced, and the associated cognitive processes.

### 2.1. Early Attentional Selectivity, Filter Tasks and Cognitive Correlates

Early attentional selectivity originates from the currently obsolete model by Broadbent [6]. According to the model, the stimuli in both the external and the internal environments are analyzed by the information processing system for their physical attributes (e.g., novelty, intensity, abrupt occurrence). Attention then acts as a filter and selects stimuli based on these physical attributes.

According to the current concept of early attentional selectivity (Table 1), stimuli are analyzed through an iterative interaction between the sensory register and long-term memory; this stage involves automatic processes of the pre- or unconscious [7]. In this early stage, attentional selectivity is perceptual; perceptual conflicts are resolved at the perceptual level, and processes of attentional selectivity are quantitively represented. In event-related potentials (ERPs), attentional selectivity is represented, for example, by component amplitudes and latencies. In the brain’s oscillatory activity, attentional selectivity is represented, for instance, by component power, phase locking, or frequency locking in the resting electroencephalogram (EEG) and event-related oscillations (EROs).

Early attentional selectivity is associated with filtering and is studied using filter tasks [5,8]. One group of filter tasks requires between-channel selection (e.g., spatial selection task). Here, participants are to select and attend to a stimulus in a given channel while they ignore the stimulus at the other channel. Between-channel selection tasks are used when the focus of interest is perceptual conflict. A second group of filter tasks produces channel interference (e.g., Stroop task, dichotic listening task). Here, participants are to perform a discriminative response to one stimulus channel. However, this response is interfered with the stimulus in a competing channel. In this case, channel interference is a perceptual conflict that is represented by the prolonged latency of the earlier perceptual components. Interference may also be a response competition; in this case, prolonged latencies are displayed in motor components and overt behavioral responses (e.g., reaction time: RT).

### 2.2. Late Attentional Selectivity, Selective-Set Tasks, and Cognitive Correlates

The concept of late attentional selectivity originates from the model developed by Deutsch and Deutsch [9]. According to this model, external and internal stimuli are analyzed for physical and semantic attributes. In contrast to early selectivity, late selectivity involves response selection. Attention acts as a filter to select a functional response from various possible alternatives.

According to the current concept of late attentional selectivity (Table 1), the previously perceived stimuli are processed via integrative associational processing within relevant brain networks [7,10]. In contrast to early attentional selectivity, late selectivity involves consciously monitored controlled processing. The selectivity in task-relevant responding involves target detection and response selection. Any conflict is thus due to response competition that is also resolved at the response level. Late attentional selectivity processes are quantitively and qualitatively (structurally) represented, for example, in a changing topography of the components and the specific processes responsible for attentional selectivity [11,12,13].

Late attentional selectivity involves the formation of selective sets [5,8]. Among the most frequently used selective-set tasks are deviance tasks. Active deviance tasks (e.g., active oddball task) ask the participant to select a rarely occurring stimulus (target) and to respond to it (task-relevant response). Passive deviance tasks (e.g., passive oddball task) use the same experimental procedure, but the task is to perform an irrelevant task. Active deviance is related to active, facilitatory attention in the form of focused and selective attention. Passive deviance is related to passive inhibitory attention. It includes an involuntary orientation reaction [14] and preconscious, passive attention [15]. Other tasks involve priming tasks (e.g., auditory priming), which produce focused attention (active facilitatory attention) via a priming stimulus, and shifting tasks, where attention (both facilitatory and inhibitory active attention) has to be moved between targets [8].

## 3. Age-Related Childhood Changes in ERP Components and Attentional Processing

The time domain methodology and analysis of brain responses were first recorded by Davis [16] as evoked potentials (EPs). The summation technique strengthened the methodology (Dawson [17]) because it made the very low amplitude brain responses, which occur within the context of an irregular and large amplitude background activity, discernable. The first event-related cognitive component (P300) was recorded by Sutton and colleagues [18,19]. Early studies on cognitive processes and the development of attention used the ERP methodology, and this trend still endures [20,21]. As the following sections show, the ERP methodology is also used in most of the studies on brain responses of TDC [5,8,21,22].

### 3.1. Early Stage of Attentional Processing: Filtering

According to a meta-analysis by Verhaeghen and De Meersman [23], the delay that channel interference produces (the Stroop effect) is a typical cognitive event that stays invariant throughout adulthood [24]. In TDC, there is a strong Stroop effect. In neuropsychological tasks, this effect is represented in longer response durations [25]. However, the ERP components represent the Stroop effect in large amplitudes [26]. These amplitudes decrease throughout childhood (5–12 years) and stabilize at the adult level around 12 years of age [24].

When attention is exogenously cued to relevant aspects of the stimulus, children’s performance reaches that of adults [27]. This finding shows that channel interference, which component amplitudes represent, is due to perceptual conflict, and the filter operates at the perceptual stage [28]. Inefficient suppression of inappropriate responses is a characteristic of young children. The Stroop effect is demonstrated by a long latency of the readiness potential (RP) and, in behavioral studies, a long RT [29]. In this case, the locus of the filter is post-perceptual; the filter is operating at the response stage.

When five-year-old children perform between-channel tasks, they respond with the attention-related N2b and P3b components. This is regardless of whether the stimulus is task relevant (to be attended) or task irrelevant (to be ignored [30]). This finding shows that TDC can detect stimuli but cannot filter out and selectively respond to the relevant stimulus. Efficient channel selection becomes observable in 7–9-year-old children [31], becomes possible around 12 years of age, and continues development until 17 years of age [32]. Several ERP components serve as markers of the development of efficient channel selection and different types of attention [33,34,35]. Two markers show latency variations. Latency of the frontal selection positivity (FSP) represents an early selection between relevant and irrelevant channels. Further processing of the attended channel is represented in the latency of N2b. A third marker shows amplitude variations in the early part of the processing negativity (PN) [36]. Age-related amplitude variations in early PN represent the development of stimulus comparison and selection.

### 3.2. Late Stage of Attentional Processing: Formation of Selective Sets

The most widely used selective-set tasks of late attentional selectivity are the active and passive oddball (OB) tasks. In adults, the prominent attention-related component of the active OB (OB-a) task is P3b [18,37,38]; that of the passive OB (OB-p) task is mismatch negativity (MMN; [39]).

#### 3.2.1. Findings on Active Deviance Detection

Attention-related ERPs to active deviance detection start in adults with N2, an early selection component. Development is predominantly represented by decreasing latencies between 5–6 and 22 years of age [40,41]. Another developmental representation is the topographical spread of N2. The diffuse representation up to 10–14 years of age evolves to a frontal distribution starting at 17 [41]. According to these N2 findings, the speed by which stimuli are discriminated and compared increases with age, and the cortical spread of the component becomes focalized to the frontal sites.

Another attention-related ERP component in adults is P300 [42,43]. P300 appears as P3a or P3b. In infancy (4–7 months of age), precursors of P3b are two late components. The first is a central–frontal negative component (Nc; onset 140–160 ms, duration 700 ms). Age-related increases in Nc amplitude represent the detection of and attention to deviant stimuli. The second precursor is a distributed late positive wave (Pc/PSW; onset 200–1000 ms, duration 1300 ms). Age-related increases in Pc amplitude represent the extent of memory encoding [44,45].

The third late component is the precursor of the frontally obtained adult P3a [43]. In adults, P3a is an orientation response to novel events and is responsible for novelty detection. It is thus obtained in response to novel, intense, or unexpected events or events that are significant to the species or the individual [37,46,47]. The P3a precursor, the negative slow wave (NSW) becomes apparent at around two years of age [48]. Its amplitude decreases from childhood, and its latency decreases up to seven years of age [11,48,49]. Nevertheless, P3a (of the N2-P3a complex) continues to display age-related changes within the 8–28 age range [50]. Increasing P3a amplitudes and decreasing latencies indicate faster processing of novel events and improved cognitive control.

The relation between P3b latency and information processing speed is a consistent finding [47]. From a young age, latencies progressively decrease until they reach adult levels at 22–25. Findings on P3b amplitude, however, do not make a general conclusion feasible. There are reports about amplitude increases until 21 [47] or 15 years of age [48] and increasing amplitudes in the frontal lobes but not in the parietal lobes, where amplitudes are similar to those of adults [51].

#### 3.2.2. Findings on Passive Deviance Detection

The composition of the stimuli (frequent standards and infrequent deviants that are serially presented in random order) in the OB-p task is identical to that in OB-a; the difference is in the task the participant is asked to perform. In OB-a, the task is to respond to a relevant stimulus (e.g., press a button, count). In OB-p, the task is to perform an irrelevant task throughout the stimulus presentation. The OB-p task produces the mismatch negativity (MMN) component in adults. The component is a frontally distributed negative component (100–300 ms), and it is obtained mainly in response to auditory stimulation (e.g., frequency and tone deviances). MMN is produced by repetitive stimulation; frequent repetition of the standard stimulus makes the sensory representation of the standard stimulus precise, as a result of which detection of even a slight difference in the deviant stimuli becomes possible. This deviance or mismatch detection is automatically performed in the preconscious [39].

However, many of the findings on the age-related changes in MMN latency and amplitude are inconsistent. Reported findings on age-related latency changes include 240 ms in newborns, 207 ms in 8-year-old children, and 140 ms in adults [52], as well as a latency decrease only between 3 and 14 years of age [53] and a stable latency throughout childhood and adulthood [36,54]. Age-related amplitude changes include almost all of the possible findings: large amplitudes throughout childhood [55], decreasing amplitudes from younger to older children [36], and a stable amplitude throughout childhood and adulthood [56].

## 4. A Bidirectional Serial Processing: Bottom-Up with Top-Down Processing

According to the previous findings, the onset of the development of attention-related components might be as early as 4–7 months of age/infancy (e.g., precursors of P300) or two years of age (e.g., P3a), but the more common initiation is around 5–7 years of age/middle childhood (e.g., N2b of between-channel selection). Depending on the type of ERP component, latencies may attain adult levels as early as seven years of age/middle childhood (e.g., for P3a), at 17 years of age/adolescence (e.g., for N2b and P3b of between-channel selection), and at 22 years of age/early adulthood (e.g., for N2 of active deviance detection) (Section 3.1 and Section 3.2).

Broadbent’s [6] theory assumes that attentional selectivity (or the filter) operates upon physically analyzed stimuli, and this early selectivity is demonstrated with filter tasks (e.g., between-channel and channel interference tasks). According to its current extensions, this is a bottom-up process. According to the theory of Deutsch and Deutsch [9], attentional selectivity (filter) operates upon semantically analyzed stimuli, and according to its current extension, the process is a top-down one. This early selectivity is demonstrated with the selective-set tasks (e.g., deviance detection tasks and target search tasks) [8].

According to the review article by Karakaş [8], age-related changes in TDC occur in both the early (findings of studies that use the various filter tasks; Section 3.1) and the late stage (findings of studies that use the selective-set tasks; Section 3.2) of attentional processing. The consistently obtained findings are quantitative and occur mainly upon component latencies. Decreasing latencies show that, as children age, they become faster and more efficient at perceptual analysis and filtering; early attentional selectivity that involves bottom-up processing is enhanced. Age-related changes in late attentional selectivity involve top-down processing, represented in the speed and efficiency of target detection, classification, and response selection.

Amplitude represents power and the associated energy; in information processing, amplitude is the biological marker of the intensity of resource allocation. Available resources and power vary according to individuals’ transitory and stable characteristics. Therefore, age-related amplitude variations are usually less consistent in TDC than in latency. However, amplitude becomes the critical index under sub-optimal stimulus conditions constraining developmental trends. In adults, task difficulty is represented in P3b amplitude. In TDC, difficulty and cognitive load are represented in the amplitude of entirely different ERP components. In children, the responsible components are the late, slow potentials, such as processing negativity (PN) [36]. In infants, detection is represented by two components unique to infancy: Nc and Pc (Section 3.2.1).

Results of studies that manipulate task difficulty/cognitive load draw attention to the interplay between early and late attentional processing stages. In adults, attentional selectivity is ordinarily perceptual (early stage). In TDC, inefficient perceptual filtering [5] due to task difficulty and cognitive load produces a shift to the later stage of information processing. Age-related changes are then represented not in perceptual but in response-related indices, such as the lateralized readiness potential (LRP), data from electromyography (EMG), behavioral RT, and response accuracy [5].

These ERP findings show that the brain does not serially operate in either a bottom-up or a top-down direction at a given time and under given conditions. Variables related to the task and/or the participant may require a shift between the mode of processing. The transition between perceptual selectivity and response selectivity, and from perceptual to response components, represents a reorganization of the cognitive processes, the ERP components, and the respective brain areas. In contrast to the age-related latency variations, which are quantitative, the reorganization of the cognitive processes and brain neuroelectric activity under task difficulty represent qualitative changes [12,13,57].

## 5. Age-Related Childhood Changes in EEG and ERO Components of Attentional Processing

As Section 2 shows, age-related changes in attentional processing have mainly used the ERP methodology, which analyzes neuroelectric activity in the time domain. The theoretical framework behind most ERP studies is the current extensions of the classical theories of attention [6,9]. These theories are based on the assumption that human information processing is a serial process that proceeds in the bottom-up or the top-down direction. Accompanying information processing is from the periphery to the center or vice versa; and from sensory/perceptual processes to successively higher cognitive functions or vice versa [7,58].

### 5.1. Oscillatory Dynamics, Principles, and Theories

An alternative noninvasive methodology analyzes resting electroencephalography (EEG) and event-related activity in the frequency or the more recently formulated time and frequency domains [59]. This approach dates back to Berger [60], who recorded human EEG for the first time and demonstrated the alpha and beta oscillations in EEG. The approach gained impact with Adrian [61], who discovered the event-related oscillation (ERO) in the gamma band.

Studies in 1980–1990 led Mountcastle [62] to pronounce a paradigm change to the oscillatory activity (EEG and EROs) for understanding brain function. This decade witnessed studies on the oscillatory dynamics of humans and many nonhuman species; developmental stages; sensation and cognition; and spontaneous, evoked, emitted, and induced brain activity [63,64]. These findings were replicated and confirmed; accumulated information led to formulating principles of oscillatory dynamics [19]. Erol Başar, who is a pioneer in the brain’s oscillatory dynamics, outlines these principles as follows [63,64,65]. The waveforms in resting EEG, EP, and ERP result from the superposition of the oscillatory components of different frequencies (the principle of superposition); each oscillation has multiple functions, and multiple oscillations represent a given cognitive function (the principle of multiplicity of function); cognitive functions are represented by oscillations that are temporally and spatially integrated within the whole brain (the principle of whole brain work). A demonstration of these principles can be found in two review papers [19,66], one on the historical background of oscillatory dynamics and the other on the theta oscillation of the brain.

Contemporary brain theories are mainly based on the principles of oscillatory dynamics [67,68]. A pioneering theory is that of Erol Başar [69]. According to the theory of whole brain work, (1) bottom-up and top-down processes occur in parallel within selectively distributed neural pathways. (2) This structural organization produces complex and dynamically changing connectivity patterns between temporally and spatially associated brain oscillations. (3) The connectivity patterns are the correlates of cognitive-affective processes of the human information processing system.

### 5.2. Attentional Processing and Resting-State EEG Components in TDC and a Clinical Model

Time domain ERP findings and formulations dominate the development of attentional processing [21,70]. However, the few studies on the oscillatory dynamics of attention provided clinically useful criteria, formed the basis of a model of attentional processing involving a wholistically functioning brain and had heuristic value for future research on the typical development of attention.

A well-established developmental finding on TPD pertains to the components decomposed from the resting EEG using frequency domain methodology [19]. At the beginning of life, EEG is dominated by spontaneously occurring slow wave activity (e.g., delta). In early childhood, theta and alpha oscillations (the faster activity) become posteriorly visible, and in time, they propagate to the anterior regions. Another fast activity, beta, becomes centrally visible, and in time, it propagates to the anterior (frontal) and the posterior regions (parietal and occipital) [71,72,73,74]. By 7.5 years of age, slow oscillations are replaced by high-frequency oscillations [20].

The spontaneously occurring beta oscillation’s relevance to conscious attentive wakefulness [60,72,75], alpha oscillation’s relevance to vigilance and selective attention [76,77], and the theta oscillation’s relevance to the differentiation of states of consciousness [78] are well-established. Some serve as criteria for the standard classification of sleep–wakefulness stages [79].

In DSM-5 [80], the basic symptom of attention deficit hyperactive disorder (ADHD) is inattention. As such, ADHD is a useful clinical model of attentional processing. Following that, a significant part of the studies on the oscillatory dynamics of attention are on children with ADHD rather than on TDC.

In ADHD, maturation of resting EEG is structurally and functionally equivalent to that in TDC, but changes occur later [20]. For example, in TDC, slow-wave ADHD dominance ceases, and high frequencies emerge at around 7.5 years of age [8]. In ADHD, this change is delayed to 10 years of age. Delayed development is the prediction of the maturational lag model of Kinsbourne [81]. This model is supported not only by oscillatory components of EEG but also by neuroanatomical and neuropsychological findings. Neuroanatomically, the most considerable cortical thickness is attained in TDC at 7.5, but in ADHD at 10.5 years of age [82,83]. When different forms of attention are behaviorally assessed using relevant neuropsychological tests, children with ADHD reach the level of age-matched TDC around 12 years of age [25].

However, the maturational lag model provides only a partial explanation; in addition to delayed maturation, there are maturational deviances in ADHD [84,85]. According to the maturational deviance model [86], the deviances are not typical to any age but are relatively permanent. These oscillatory deviances [8] consist of low relative alpha in the parietotemporal region [20,87,88]; low absolute and relative beta in the parietotemporal and frontal areas [20,89]; low absolute EEG power in the theta band [90]; and elevated absolute and relative theta in the frontal regions and the frontal midline area [20,91]. The ratio between the elevated theta and the reduced beta, the theta/beta ratio (TBR), is specific to ADHD, and it thus serves as a biomarker of ADHD and as an auxiliary criterion for ADHD diagnosis [92,93]. Deviant patterns are also revealed in neuropsychological test scores. Using a battery of tests on different aspects of attentional processing, Erdoğan Bakar and Karakaş [25] provided evidence for both the maturational delay and the maturational deviance model that persisted throughout the studied age range (6–12 years). The test scores represent atypical information processing characterized by non-attentional factors. Similarly, the response strategies were based on impulsive and/or hyperactive behavior rather than attentional processes.

Additively, structural and functional resting-state deviancy is coupled in ADHD with deviant or reduced connectivity patterns. Per the whole brain work theory [69], high global integrity typically characterizes the brain nodes [94]. In ADHD, integrity is impaired both structurally and functionally [95]. This impairment is due to a specific redistribution of the regional nodes over the resting-state network, resulting in high local clustering and low global integrity [96,97].

Reduced differentiation and specialization in cortico-cortical circuits are associated with clusters of deviant oscillatory activity [72,75,98]. The cortical hypoarousal cluster of ADHD is characterized by elevated frontal theta, reduced delta, and reduced and deficient beta (hence the elevated TBR). The cortical hyperarousal cluster is characterized by elevated frontal beta, deficient delta, and alpha. The maturational lag cluster is characterized by elevated delta and theta and reduced and deficient beta. The last cluster is characterized by increased frontal beta. These clusters are not only neuroelectric entities; they are accompanied by specific behavior patterns typical to children with ADHD [99].

### 5.3. Attentional Processing and EROs in TDC and ADHD

The typical development of attentional processing is correlated with a quantitative change involving a decremental trend in the latencies of ERP components. This trend indicates that TDC gradually assumes the adult levels of early and late attentional selectivity. In ADHD, however, attentional processing is represented in component amplitudes. This is a quantitative change, but unlike the consistently decremental trend in component latencies of TDC, the amplitude variations are atypical. They apply to early selection processes, such as perceptual analysis, filtering, and automatic processing; and they represent involuntary attention, orienting disorder, and impairment in selective, focused, and sustained attention [66]. Atypical amplitude variations also apply to late selection processes, such as target detection, controlled processing, and response-related processes, and they represent filtering deficits, selectivity that is principally guided by stimulus salience, and deficits in attentional resource allocation [8].

The power and energy of the waveform amplitudes represent resource availability in information processing [8]. A heuristic from the preceding sections on the oscillatory dynamics of ADHD versus TDC may be the following. The experimental tasks are difficult for children with ADHD, so they invest whatever resources they possess. Despite this effort, they continue displaying the impairments of the disorder and perform poorly on attention-related tasks.

#### 5.3.1. Event-Related Gamma Oscillation: Findings and Theory

The gamma band response (GBR) was discovered later than the other frequency bands, and unlike them, it was found as an event-related component [61]. The GBR was considered among the brain’s basic operating rhythms [62,63]. Gamma also became the most frequently studied oscillation in ADHD because gamma band oscillation represents the facilitatory and inhibitory attentional processes that are clinically impaired in ADHD [80] and it modulates dopamine polymorphisms that affect the heritability of ADHD [100].

In line with the multiplicity of function principle, the GBR represents different processes [101]. In the early time window (0–150 ms), a phase-locked GBR takes place in bottom-up processing in sleep and wakefulness [78,102]. This early gamma is associated with sensory processing [103]. However, early gamma response is also associated with higher cognitive functions, such as gender specificity [104] and neuropsychological test scores [105]. A nonphase-locked GBR of the late time window (130–400 ms) is also associated with higher cognitive functions [101].

A third group of early studies on adults asserted that the GBR is related to attentional processing [106,107,108]. In line with the ‘principle of superposition,’ event-related gamma is superposed on event-related theta. During bottom-up processing, the gamma/theta superposition represents the allocation of attention. During top-down processing, the gamma/theta superposition represents the activation of memory stores [105,109,110,111,112,113].

To our knowledge, Yordanova and colleagues conducted the only research on TDC’s GBR [114,115,116]. During a between-channel task, TDC (9–12 years of age) responds to the attended stimuli with large, synchronized, and phase-locked (120 ms post-stimulus) GBR. Gamma is observed in the left frontocentral area. Under the same experimental condition, the GBR of older TDC (3–16 years of age) was ipsilateral to the side of stimulation.

Hermann and colleagues [111] integrated previously mentioned processes (bottom up and top down) and functions (attention, sensation, perception, higher cognitive functions) that operate via the GBR within the context of the match and utilization model (MUM). According to the model, the first phase is a bottom-up process. Evoked and phase-locked GBR in the first part of the early time window (0–150 ms) represents the allocation of resources to rapid processing and classification of input. Stimulus features are analyzed and integrated; sensation is transformed into perception. In the latter part of the early time window, processing is top-down. Perceived content is compared with existing memory representations. Attention modulates matching, and the GBR is enhanced with a match or the anticipation of a match [110,112,117].

An induced, nonphase-locked GBR characterizes the second phase in a later time window (approximately 200 ms following stimulus onset). The induced GBR represents the utilization of the information. In this phase, information is stored, and attention is redirected. The induced GBR of the second phase is recorded from highly distributed networks, indicating connectivity between selected areas of the brain [110,112].

Findings on ADHD support the assumptions of the MUM. In the first part of the early phase, episodes of inattention are accompanied by reduced activity, showing impairment of early automatic processing, which includes extraction, analysis, and integration of sensory information [118,119,120]. A stronger phase locking and synchronization shows alteration in early auditory processing [116].

In the second part of phase one, stimuli are encoded for later stimulus recognition and represented in task-related GBR enhancement. However, in ADHD, the later recognition performance does not accompany this enhancement, indicating that GBR enhancement is associated with a general activation of processing resources not specific to the relevant stimuli [121]. The GBR to known stimuli is not enhanced. According to the MUM, this represents an inability to allocate processing resources or a failure to access and match stimuli with the relevant memory representation [122]. Atypical or impaired processing leads to impaired stimulus filtering. Then, attention cannot be shifted from the irrelevant input or focused on the relevant input [123,124]. The mainly quantitative problems in the early stage lead to inattention and distractibility, leading to atypical involuntary and orienting attention and impaired active attention consisting of selective, focused, sustained, and inhibitory attention [123,124].

Impairments in the first phase influence the processes in the second phase, and children with ADHD make compensatory attempts to accomplish the early-phase processes [21]. The amplitudes of components representing late processing (e.g., P3b, FSP SN) are reduced [122,125,126,127,128]. The qualitative problems in the late stage of attentional processing mainly lead to difficulties in target detection, response-related processes, and controlled processing [8].

#### 5.3.2. Event-Related Theta Oscillation: Findings and Theory

Theta was discovered by Jung and Kornmüller [129] in the rabbit hippocampus. Later research found that theta oscillation plays a role in many cognitive processes such as sensation, perception, learning, memory, navigation, and motor processes [14,130,131,132,133,134,135,136,137,138]. As such, theta is an excellent demonstration of the ‘multiplicity of function principle’ of oscillatory dynamics [139,140,141,142].

The most recent review [66] systematizes the currently known functions and structures of theta under the cortico-hippocampal interplay model. The network where this interplay occurs is highly interconnected. Consequently, information travels over a densely interconnected, highly synchronized system via the theta oscillation. This architecture bestows theta with the many cognitive-affective correlates, one of which is attention. In ADHD, where connectivity is impaired [95], the corticocortical theta circuits are characterized by high clustering and low global integrity, resulting in reduced cortical differentiation and specialization [143].

The primary oscillation that composes the novelty N2-P3a waveform is theta [144]. Wienke and colleagues [50] studied the connectivity strengths through inter-site coherences between 48 pairs of electrodes in participants 8–28 years of age. Age and theta phase coupling were linearly related, showing that theta is a maturation marker from late childhood to young adulthood. Enhanced connectivity strengths were displayed in the short-range frontocentral and the long-range anteroposterior connections. These findings support those of Barry and colleagues [143] and indicate that typical maturation includes increased integration of frontal brain activity (seed electrodes were Fz, FCz, and Pz) within a widely distributed neural network (the long-range anteroposterior connections).

Research [43,76,77,109,141,145,146,147,148] indicates that spontaneous theta in the resting EEG is responsible for arousal. Event-related theta or the superposition of theta with oscillations in other frequency bands (e.g., theta superposed on the delta at P3b latency, theta superposed with alpha at 100 ms latency) are responsible for all sorts of attention that filter and selective-set tasks produce (Section 2). These include active and passive attention that the active deviance and passive deviance tasks, respectively, produce; facilitatory attention (selective, focused, and sustained attention) and inhibitory attention (as in the Stroop task); early attentional selectivity at approximately 150 ms (as in the filter task); and late attentional selectivity at approximately 300 ms (as in the selective-set tasks).

During active and passive attention, adults display an enhanced and strongly phase locked early theta response (0–300 ms) and a nonphase-locked late theta response (300–600 ms). Early theta is associated with a N200 component; late theta is associated with a P3b-like P400–700 component [115]. The high-amplitude early theta response of TDC displays weak phase locking. As children age, phase locking gets more robust, and the latency of the theta response decreases [114].

However, despite its relevance to almost all aspects of adult attentional processing, conclusive research was not devoted to developing attention in TDC. Theta may be a possible marker of ADHD or may serve as an auxiliary device for diagnosis. This issue did not capture the attention of the scientific community.

## 6. Conclusions

The present paper reviews the development of attention via resting state EEG, ERPs, and EROs in children (6–12 years of age). Findings on the earlier stage of attentional processing were studied using filter tasks (e.g., between-channel and channel interference tasks). Those in the later stage of attentional processing were studied using selective-set tasks (e.g., deviance detection, priming). Findings were discussed within serial (bottom up, top down), serial–bidirectional, and holistic–integrative information processing. Qualitative changes that involve a transition from early to late attentional processing were demonstrated in experimental designs that use difficult tasks with high cognitive load. Another approach involved using ADHD as a clinical model of attentional processing.

The review highlights the following issues:

- The task that the experiment uses determines the type and stage of attentional processing. Early attentional selectivity is studied with the filter tasks (between-channel selection, channel selectivity) and late attentional selectivity with the selective-set tasks (active deviance detection, passive deviance detection, target search, attention priming, shifting).
- Attention has an independent and differential effect on all other aspects of information processing. Thus, it deserves scientific interest as a cognitive phenomenon that must be explained and a confounding variable that must be controlled.
- Typical development of attention is quantitatively represented in latency variations of ERP components. However, attention is qualitatively represented when a task is difficult. Late attentional processing becomes operational, late ERP components appear, and components vary in amplitudes.
- The resting EEG quantitatively represents the typical development of attention, where a transition from a dominance of the slower oscillations to the faster ones occurs.
- ADHD is a useful clinical model for studying attention since the basic and most frequent symptom of the disorder is inattention.
- In ADHD, the resting EEG development follows the TDC trend, albeit at later ages. Such a delay also applies to neuroanatomical development and age-related changes in neuropsychological test scores. These findings support the maturational lag model.
- Some of the ADHD findings indicate atypical and relatively permanent organization of attentional processes and the respective brain areas. These findings support the maturational deviance model.
- ADHD is also a result of deviant connectivity patterns between brain areas. The structural and functional impairment is associated with a nodal redistribution of the default network that involves high local clustering and low global integrity. Findings indicate four types of EEG clusters and the accompanying behavioral patterns resembling the inattention, impulsivity, and hyperactivity symptoms of ADHD.
- According to the MUM, attentional processing is represented by variations in the gamma oscillation within the context of a wholistically functioning brain.
- Studies on clinical models (e.g., ADHD) may produce groundbreaking findings and formulations on the nature of attention.

Time domain ERP findings dominate the literature on typical attentional development. This approach largely ignores the holistic–integrative brain function and implicitly assumes a serial processing of information in either the bottom-up or the top-down direction. Although less abundant, the frequency domain approach involving the brain’s oscillatory activity could provide biomarkers and auxiliary criteria for ADHD diagnosis. The match and utilization model demonstrated attentional processing within a selectively distributed parallel processing brain system that used gamma oscillation. The multifunctional theta oscillation has the potential to explain attentional processing and its different components. Future studies focusing on the typical development of attention may invest greater interest in oscillatory dynamics and benefit from its methodology and theoretical background.

## Figures and Tables

**Table 1:** Experimental Tasks, Types of Interference and Attention According to Stages of Attentional Selectivity and Information Processing.

Stage of Attentional Selectivity	Type of Information Processing	Relevant Experimental Tasks	Interference	Type of Attention
Early Attentional Selectivity	Filtering	Between-channel tasks	Perceptual conflict	Active/facilitatory attention
		Channel interference tasks	Perceptual conflict, response competition	Active/facilitatory and inhibitory attention
Late Attentional Selectivity	Selective-Set	Active deviance detection tasks	Target detection with response competition	Active/facilitatory attention
		Passive deviance detection tasks		Passive/facilitatory attention
		Target search tasks		Active/facilitatory attention
		Attention priming tasks		Active/facilitatory attention
		Attention shifting tasks		Active/facilitatory and inhibitory attention
